# Reprogramming of pathogenic strategies in *Fusobacterium nucleatum*: a study based on an *in vitro* infection model

**DOI:** 10.3389/fcimb.2026.1719385

**Published:** 2026-03-31

**Authors:** Li Ma, Xiaoyu Du, Chenyu Pang, Xuan Han, Jianquan Zhang, Xiaowei Guo, Ke Pu

**Affiliations:** 1 Department of Preventive and Pediatric Dentistry, School and Hospital of Stomatology, Tianjin Medical University, Tianjin, China; 2 Department of Preventive and Pediatric Dentistry, Tianjin Medical University School and Hospital of Stomatology & Tianjin Key Laboratory of Oral Soft and Hard Tissues Restoration and Regeneration, Tianjin, China; 3 Department of Pediatric Dentistry, Anyang Hospital of Stomatology, Henan, China; 4 Baodi Hospital, Tianjin Medical University, Tianjin, China; 5 Department of Neurosurgery, Tianjin Huanhu Hospital, Tianjin, China

**Keywords:** Blood-brain barrier, brain microvascular endothelial cells, drug susceptibility, *Fusobacterium nucleatum*, intracranial infection, proteomics

## Abstract

**Background:**

*Fusobacterium nucleatum* is a Gram-negative obligate anaerobic bacterium with trans-organ pathogenicity. It serves as a crucial pathogen causing intracranial anaerobic infections and is closely associated with the occurrence of brain abscess. However, the mechanism by which *Fusobacterium nucleatum* crosses the blood-brain barrier and induces intracranial infection remains unclear.

**Methods:**

In this study, *Fusobacterium nucleatum* clinical strain isolated from the brain abscess was identified and characterized. Its growth characteristics and biofilm-forming capacity were compared with those of the ATCC 25586 standard strain. Furthermore, the adhesive and invasive capabilities of both strains towards oral epithelial cells and brain microvascular endothelial cells were assessed. An *in vitro* BBB model was constructed using a brain microvascular endothelial monolayer to examine the effects of bacterial infection on the expression of tight junction proteins (occludin, Claudin-5, ZO-1). Finally, antimicrobial susceptibility testing combined with proteomic techniques was employed to analyze the antibiotic resistance profiles and differentially expressed proteins of the strains.

**Results:**

One clinical strain was successfully isolated and identified from specimens of 7 brain abscess patients. Compared to the standard strain, the clinical isolate demonstrated a slower growth rate, reduced biofilm formation, and diminished adhesion and invasion capabilities against both oral epithelial cells and brain microvascular endothelial cells. The two strains exhibited significantly distinct regulatory patterns on the tight junction protein expression in brain microvascular endothelial cells. Proteomic analysis revealed extensive protein reprogramming in the clinical strain, characterized by the upregulation of proteins involved in metabolic pathways and immune evasion. Altered antimicrobial susceptibility in the clinical strain correlated with differential expression of proteins such as ribosomal components and efflux pump proteins.

**Conclusion:**

*Fusobacterium nucleatum* can specifically adhere to and invade brain microvascular endothelial cells, disrupting their tight junctions. Through proteomic reprogramming, *Fusobacterium nucleatum* enhances its metabolic adaptation and immune evasion capabilities to adapt to changes in the intracranial microenvironment and establish persistent infection, thereby achieving a strategic shift from barrier penetration to intracranial colonization.

## 1 Introduction

*Fusobacterium nucleatum* (*F.n*) is a gram-negative, obligate anaerobic bacterium and a core colonizer of the human oral microbiota. It can coaggregate with other oral microorganisms, such as Streptococcus and Actinomyces species, via surface adhesins, functioning as a bridging organism in the formation of dental plaque biofilms (Han, 2015; Chen et al., 2022; Jiang et al., 2025; Tanaka et al., 2025). This role is closely associated with the development of various oral infectious diseases, including periodontitis and periapical periodontitis (Yu et al., 2020). Recent epidemiological and molecular pathological studies have confirmed that *F.n* can translocate to distant sites via hematogenous dissemination or lymphatic spread, contributing to the pathogenesis of a range of systemic diseases. These include colorectal cancer (Denes and Barraud, 2016), inflammatory bowel disease (Xiang et al., 2025), cardiovascular diseases (Chen et al., 2022), Alzheimer’s disease (Vander Haar et al., 2018), and adverse pregnancy outcomes (García-Rios et al., 2026), demonstrating its significant trans organ pathogenicity. Meanwhile, *F.n* can evade elimination by the host innate immunity through multiple mechanisms, such as interfering with pattern recognition receptor signaling, inhibiting phagocytic killing, targeting inhibitory receptors on immune cells, and remodeling the local microenvironment (Quah et al., 2014; Chen et al., 2018; Gur et al., 2019; Engevik et al., 2021; Jiang et al., 2025). These immune evasion mechanisms underpin its ability to achieve long-term colonization and pathogenicity within the host.

Brain abscess is a suppurative infection of the central nervous system characterized by insidious onset and rapid progression. Its annual incidence ranges from 0.3 to 1.3 per 100,000 individuals, with a mortality rate as high as 10%-24% (Pereira et al., 2022). It is frequently complicated by severe conditions such as hydrocephalus and brain herniation (Brouwer and van de Beek, 2017). Even with standardized treatment, a high disability rate remains, posing a significant challenge in clinical management. Traditionally, aerobic bacteria such as Streptococcus and Staphylococcus species have been considered the predominant pathogens in brain abscess (Hall and Mesfin, 2025). With the widespread clinical application of molecular detection technologies such as next-generation sequencing (NGS), the proportion of anaerobic bacteria among brain abscess pathogens has increased year by year, and they have become important etiological agents of intracranial purulent infections. Recently, multiple cases of brain abscess caused by *F.n* as a single pathogen or in mixed infections have been reported internationally, confirming that this bacterium is a crucial pathogen responsible for intracranial anaerobic infections. However, *F.n* requires stringent culture conditions: it is a strict anaerobe with complex nutritional requirements, leading to an extremely low success rate of isolation and culture from the pus of brain abscess lesions. This issue has long hindered the research progress on the pathogenic mechanisms linking *F.n* to brain abscess.

The barrier system of the central nervous system consists of the blood-brain barrier (BBB), blood-cerebrospinal fluid barrier (BCSFB), and arachnoid barrier, which collectively serve as critical structures for defending against pathogen invasion and maintaining homeostasis of the internal environment within the CNS (Abbott et al., 2010; Kaya and Ahishali, 2021). Among these, the blood-brain barrier, characterized by tighter cellular junctions and more stringent restrictions on substance permeability, represents the primary barrier preventing pathogens from entering the brain parenchyma (Redzic, 2011). The BBB is primarily composed of brain microvascular endothelial cells, astrocytes, pericytes and neuronal projections which are in contact with astrocyte end feet’s (Kaya and Ahishali, 2021; Dotiwala et al., 2025). The core of its physical barrier relies on tight junctions between brain microvascular endothelial cells, with the expression and dynamic regulation of tight junction proteins such as occludin, Claudin-5, and ZO-1 directly determining the permeability and defensive function of the BBB (Lochhead et al., 2020). Although *F.n* has been confirmed to be associated with brain abscesses, the molecular mechanisms by which this bacterium breaches CNS defense barriers such as the blood-brain barrier, rapidly adapts to the unique intracranial microenvironment characterized by hypoxia and high immune pressure, evades local immune recognition, and achieves colonization and proliferation remain unclear. Furthermore, systematic experimental studies on the adhesion and invasion characteristics of *F.n* toward brain microvascular endothelial cells, as well as its regulatory patterns on tight junction proteins of the blood-brain barrier, are currently lacking.

To investigate the biological mechanisms underlying intracranial infections caused by *F.n*, this study employed a two-strain system comprising the standard strain and the clinical strain. First, the clinical strain derived from brain abscess were isolated and identified. Subsequently, the biological characteristics of both strains, such as growth and biofilm formation, were compared. Their adhesion and invasion abilities, as well as their regulatory effects on tight junction proteins in blood-brain barrier endothelial cells, were assessed. Combined with antimicrobial susceptibility testing to analyze the resistance profile of the clinical strain, differentially expressed proteins were screened using proteomics techniques. This study aims to provide deeper insights into the pathogenic mechanisms of *F.n* in intracranial infections.

## 2 Materials and methods

### 2.1 Experimental materials

#### 2.1.1 Strain sources

The *F.n* standard strain ATCC 25586 was purchased from the American Type Culture Collection (ATCC). The clinical strain was isolated from the pus sample of a brain abscess patient and identified as *Fusobacterium nucleatum* by NGS. The collection of brain pus specimens and strain culture in this study were performed in accordance with the following criteria: patients voluntarily signed informed consent, NGS confirmed the presence of *F.n* infection with a relative abundance of no less than 30%, and patients did not receive any antibiotic treatment within one month before admission. As brain abscess has an insidious onset and rapid progression, most patients were not aware of the bacterial infection at the initial stage and therefore did not use antibiotics before medical consultation. This inclusion criterion did not affect clinical diagnosis and treatment, nor did it significantly reduce the number of collected samples. And the patients had no severe systemic underlying diseases. Brain pus specimens were collected during brain abscess surgery after obtaining informed consent from the patients. The whole experiment process had been approved by the Institutional Review Board and the Ethics Committee at the Hospital of Stomatology, Tianjin Medical University (No. TMUhMEC20230402).

#### 2.1.2 Cell lines

Human gingival epithelial cells (KB) were purchased from the Cell Bank of the Chinese Academy of Sciences (Catalog No. TCHu73). Mouse brain microvascular endothelial cells (bEnd.3) were purchased from ATCC (Catalog No. CRL-2299).

### 2.2 Isolation, culture and identification of clinical strain

#### 2.2.1 Isolation and culture

Suspected strains were isolated from brain pus specimens via anaerobic culture and subculture. Monoclonal strains were obtained after purification and three consecutive passages.

#### 2.2.2 Biochemical identification and molecular identification

Biochemical identification was performed in accordance with the criteria for the genus Fusobacterium documented in Bergey’s Manual of Systematic Bacteriology, and with reference to WS/T 805-2022: Basic Technical Standard for Clinical Microbiology Laboratory (National Health Commission of the People’s Republic of China). The fermentation abilities of the strains for carbohydrates including glucose, lactose and sucrose were examined using bacterial micro-biochemical identification tubes, while the production of indole and hydrogen sulfide was also detected.

Total genomic DNA of the strains was extracted by the phenol−chloroform method. PCR amplification was carried out with 16S rDNA as the target gene, and the amplification products were verified by agarose gel electrophoresis before sequencing.

### 2.3 Determination of growth status and biofilm formation ability of clinical strain

The two strains were inoculated into liquid medium separately and anaerobically cultured at 37 °C. Samples were collected every 2 hours to measure the absorbance at 600 nm. The experiment was performed in triplicate. Growth curves were plotted to analyze the growth and proliferation of the strains.

The biofilm formation ability was determined by crystal violet staining. Bacterial suspension was inoculated into 96-well plates at 100 μL per well and anaerobically incubated at 37 °C. At different time points, the samples were stained with 0.1% crystal violet and dissolved with 33% glacial acetic acid, and the absorbance at 590 nm was measured. The experiment was repeated three times, and biofilm formation curves were plotted.

### 2.4 Antimicrobial susceptibility test

Antimicrobial susceptibility testing was performed according to the standards and protocols of CLSI M11 and M100 (Institute CaLS, 2018; Institute CaLS, 2024).

The minimum inhibitory concentration (MIC) was determined using the two−fold dilution method. Based on the results of preliminary experiments, minocycline (MNO, 250 μg/mL), clindamycin (CLI, 250 μg/mL), gentamicin (GEN, 25 mg/mL), and cephradine (CED, 2.5 mg/mL) were added to 96−well plates and subjected to serial two−fold dilution. The adjusted bacterial suspension (2×10^8^ CFU/mL) was added to each well, followed by anaerobic incubation at 37 °C for 48 h. The lowest drug concentration with no visible bacterial growth was recorded as the preliminary MIC value, and the experiment was performed in triplicate. A Probit regression model was established with the logarithm of drug concentration as the abscissa and the bacterial growth inhibition probability as the ordinate. The drug concentration corresponding to a 50% inhibition probability was calculated by interpolation and defined as the definitive MIC value.

The Kirby-Bauer disk diffusion method was employed to visually assess the inhibitory effects of the antibiotics. Based on the MIC results, the antibiotic concentrations were adjusted with deionized water as follows: MNO 25 μg/mL, CLI 25 μg/mL, GEN 25 mg/mL, and CED 2.5 mg/mL. A bacterial suspension with a concentration of 2×10^8^ CFU/mL was evenly spread on the surface of a solid culture medium, followed by the application of antibiotic susceptibility disks containing the corresponding antibiotic concentrations. After anaerobic incubation at 37°C for 24 hours, the diameters of the inhibition zones were measured.

### 2.5 Inhibition of cell proliferation by *F.n* on KB cells and bEnd.3 cells

KB cells and bEnd.3 cells were seeded into 96-well plates at a density of 1×10^4^ cells per well. After 24 h of culture, bacterial suspensions were added at MOI = 10:1 and 100:1, followed by co-culture at 37°C with 5% CO_2_ and 21% O_2_ (normoxia) (Ohkusa et al., 2022; McGregor and Wolthers, 2025). Untreated cells were used as the control group. At 3, 6, 18 and 24 hours post-infection, Cell Counting Kit-8 (CCK-8) reagent was added. Following incubation, the absorbance at 450 nm was measured, and cell viability was calculated according to the formula. The experiment was independently repeated three times to assess the inhibitory effect of the bacteria on cell proliferation and to determine the optimal MOI.

### 2.6 Adhesion and invasion ability assays of *F.n* to KB and bEnd.3 cells

The plate counting method was used. The two cell lines were seeded into 6-well plates at a density of 5×10^5^ cells per well, respectively. After 24 h of culture, bacterial suspensions were added at an MOI of 10:1 (this multiplicity of infection was confirmed to exert no significant inhibitory effect on cell proliferation in preliminary experiments), and co-culture was performed at 37°C under 5% CO_2_ and 21% O_2_.

Adhesion assay: After 1 hour of co-culture, non-adherent bacteria were removed by washing. The cells were then lysed, and the resulting suspension was serially diluted and plated onto culture medium. After anaerobic incubation for 7 days, bacterial colonies were counted.

Invasion assay: After 4 hours of co-culture, the medium was replaced with fresh medium containing 10 µg/mL metronidazole (preliminary experiments confirmed this concentration completely eradicated extracellular bacteria) for an additional 1 hour of incubation. Subsequent steps were identical to those described for the adhesion assay.

The adhesion rate and invasion rate were calculated using the following formulas:


$$\begin{array}{c} \text{Adhesion rate }\left(\%\right)=(\text{Number of adherent bacterial colonies}/\\ \text{Total number of bacterial colonies added})\times 100\%. \end{array}$$

$$\begin{array}{c} \text{Invasion rate }(‰)=(\text{Number of invasive bacterial colonies}/\\ \text{Total number of bacterial colonies added})\times 1000‰. \end{array}$$

Each experiment was performed in triplicate.

### 2.7 Expression of tight junction proteins in bEnd.3 cells

#### 2.7.1 Establishment of an *in vitro* monolayer cell model

Transwell inserts (pore size 0.4 μm, Corning) were coated with a 0.1% gelatin solution and incubated overnight at 4 °C. Cells were then seeded onto the inserts at a density of 1×10^5^ cells/cm^2^ and cultured in DMEM supplemented with 10% fetal bovine serum for 5–7 days.

TEER measurement: TEER was measured daily for 5 days using a voltmeter-ohm meter and an electrode system to assess barrier integrity. The measured TEER values were corrected by subtracting the background TEER of cell-free (blank) Transwell inserts. The model was considered successfully established when a stable TEER value within the range of 15 Ω·cm² to 35 Ω·cm² was achieved (Sun et al., 2022).

Permeability of sodium fluorescein: After the TEER values stabilized, fluorescein sodium (5 μg/mL) was added to the medium in the apical chamber. Following incubation for 1 hour, the medium from the basal chamber was collected. The mean fluorescence intensity was measured using a fluorescence microplate reader at excitation/emission wavelengths of 460 nm/515 nm. The permeability of the model to fluorescein sodium is expressed as the permeability coefficient (Liu et al., 2012). Permeability coefficient (P, cm/s) = [volume of basolateral chamber/(surface area of Transwells x the initial concentration of apical chamber)] × [the diffusion concentration of basolateral chamber/diffusion time].

#### 2.7.2 Establishment of an *in vitro* model of *F.n* infection in brain microvascular endothelial cells

Experimental groups were established as follows: the standard strain group, the clinical strain group, a bacterial negative control group using Streptococcus salivarius, and a blank control group. Using the *in vitro* bEnd.3 cell barrier model with established barrier integrity, the medium in the apical chamber was replaced with DMEM containing different bacterial strains at an MOI of 10:1, while the basal chamber contained bacteria-free DMEM. For the blank control group, both chambers contained an equal volume of DMEM without bacteria. Each group was set up with three replicate wells and placed in an incubator for co-culture at 37°C with 5% CO_2_ and 21% O_2_.

At 1 h, 4 h, and 6 h post-infection, the Transwell inserts were collected, washed, and lysed on ice using lysis buffer containing PMSF and protease inhibitors. The lysates were centrifuged, and the supernatants were collected. The expression levels of tight junction proteins (occludin, Claudin-5, and ZO-1) in the bEnd.3 cells were measured according to the manufacturer’s instructions of the respective ELISA kits.

### 2.8 Proteomic analysis

#### 2.8.1 Protein extraction and quantification

Bacteria in the logarithmic growth phase (OD600 = 1.0) were collected and ultrasonicated (power 300 W, working for 3 s, interval 5 s, 30 times in total). 10% TCA was added to precipitate proteins, which were left to stand at 4°C for 2 h, then centrifuged at 12,000 rpm for 15 min. The precipitate was washed three times with cold acetone, air-dried at room temperature, and then redissolved in 8 M urea. The protein concentration was determined using the BCA method.

#### 2.8.2 Enzymatic hydrolysis and LC-MS/MS analysis

50 μg of protein was taken, reduced with dithiothreitol (DTT), alkylated with iodoacetamide (IAA), and enzymatically hydrolyzed with trypsin (enzyme:protein=1:50) at 37°C for 16 h. The peptides were desalted using a C18 column and separated using the UltiMate 3000 RSLCnano system (mobile phase A: 0.1% formic acid in water; mobile phase B: 0.1% formic acid in acetonitrile) with gradient elution: 0–5 min 2%-8% B, 5–40 min 8%-35% B, 40–45 min 35%-95% B, 45–50 min 95% B. The Q Exactive HF mass spectrometer was used for detection in data-dependent acquisition (DDA) mode (scan range m/z 350-1500, primary mass spectrometry resolution 60,000, secondary mass spectrometry resolution 15,000).

#### 2.8.3 Data analysis

Three biological replicates were established for each bacterial strain group. Database search was performed using MaxQuant (v1.6.6) against the *Fusobacterium nucleatum* UniProt proteome database (download date: October 2023). During the database search of mass spectrometry data, the false discovery rates (FDR) were set at less than 1% at both the protein and peptide levels. Differentially expressed proteins were screened using the Benjamini-Hochberg method for FDR correction (adjusted FDR< 0.05), and the final set of differentially expressed proteins was determined by combining fold change (FC > 1.5 or< 0.5) with a t-test P-value< 0.05.The Gene Ontology (GO) and Kyoto Encyclopedia of Genes and Genomes (KEGG) databases were used for functional annotation and pathway enrichment analysis of differentially expressed proteins (*p* < 0.05 was considered significant enrichment).

### 2.9 Statistical analysis

GraphPad Prism 9.0.2 software was used for data analysis. The measurement data were expressed as “mean ± standard deviation (x̄ ± s)”. Inter-group comparisons: Repeated measures analysis of variance (RM-ANOVA) was used for growth curves and biofilm curves; one-way analysis of variance (One-way ANOVA) was used for the CCK8 experiment, and Dunnett t-test was used for comparisons between the experimental group and the control group. For the colony count (CFU) data from the cell adhesion and invasion assays, compliance with a Poisson distribution was verified using the Chi-square goodness-of-fit test (*p >*0.05). Inter-group comparisons were performed using the independent samples Z-test for Poisson-distributed data. The effects of bacterial treatment groups and time points on tight junction protein expression were analyzed using two-way analysis of variance (Two-way ANOVA). The t-test was used for differential analysis in proteomics. A *p* < 0.05 was considered statistically significant.

## 3 Results

### 3.1 Specimen collection and isolation and identification of *F.n* clinical strain

**Figure 1:**
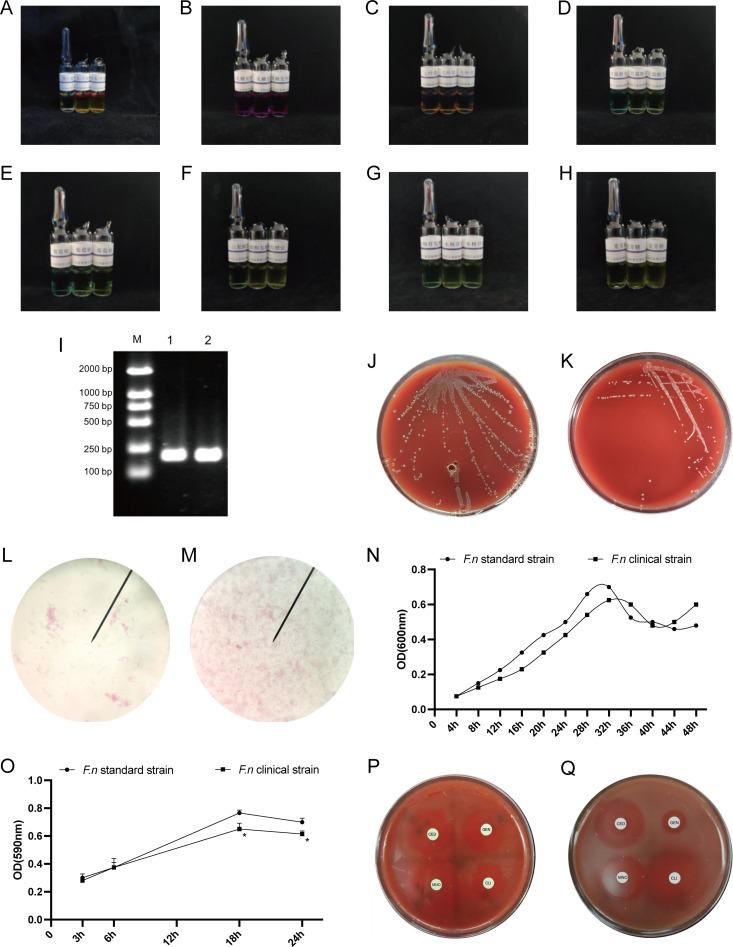
**(A–H)** were indole fermentation tube, lactose fermentation tube, esculin fermentation tube, mannitol fermentation tube, glucose fermentation tube, sorbitol fermentation tube, salicin fermentation tubes, maltose fermentation tube. From left to right, there were blank control, *F.n* standard strain and *F.n* clinical strain in each group. **(I)** PCR identification result for *F.n* standard (lane 1) and clinical (lane 2) strains, with DNA marker (lane M). **(J)** The colony morphology of *F.n* standard strain was uneven and breadcrumb-like. **(K)***F.n* clinical strain had smaller colonies and smoother morphology. **(L, M)***F.n* standard strain and *F.n* clinical strain were gram-negative with elongated bodies, pointed at both ends and filamentous under the light microscope. **(N)** The bacterial growth curves showed that in the BHI liquid medium, the growth rate of the *F.n* clinical strain was slightly lower than that of the *F.n* standard strain. The final reproduction of the two bacterial strains was almost the same. **(O)** The biofilm curves showed that the biofilm formation process was similar between the *F.n* standard strain and the *F.n* clinical strain. The final biofilm formation of the *F.n* clinical strain was lower than that of the *F.n* standard strain. The difference in the biofilm formation capacity of the two strains was statistically significant (*p* < 0.05) at the 18 h and 24 h time points. **(P)** Inhibition zones of MNO, CLI, GEN and CED against the *F.n* standard strain. **(Q)** Inhibition zones of MNO, CLI, GEN and CED against the *F.n* clinical strain.

A total of 7 pus samples from patients with brain abscess were collected and included in this study (**Table 1**). All specimens were obtained from male patients. The patients were predominantly middle-aged and elderly. The proportions of patients complicated with diabetes mellitus and hypertension were 28.57% and 57.14%, respectively. Among the 4 patients who received cerebrospinal fluid (CSF) examination, 3 patients had elevated total protein levels and leukocyte counts beyond the normal reference ranges. By means of biochemical identification and PCR detection (**Figures 1A–I**; **Table 2**), one clinical strain of *Fusobacterium nucleatum* was successfully isolated and identified from Sample 5.

**Table 1:** Clinical information of patients.

Case No.	Gender	Age	Relative *F.n* abundance (%)	Comorbidities	CSF		
					Total Protein (g/L)	WBC (10^6^/L)	Glucose (mmol/L)
1	56	M	70.18	/	0.9	10	3.24
2	56	M	99.66	Hypertension	/	/	/
3	79	M	65.69	/	/	/	/
4	85	M	46.32	Hypertension	/	/	/
5	75	M	98.42	Hypertension	0.73	2	3.91
6	63	M	63.00	Hypertension; T2DM	1.39	60	3.4
7	67	M	39.30	T2DM	1.78	1750	7.11

CSF is cerebrospinal fluid. WBC is white blood cells. T2DM is Type 2 Diabetes Mellitus. / indicates no comorbidities. Reference ranges for CSF indicators: Total Protein, 0.15–0.45 g/L; CSF WBC, 0-5×10^6^/L; CSF Glucose, 2.5–4.5 mmol/L. "/" indicates that the patient did not undergo CSF puncture. This was because the patient had developed focal lesions and was diagnosed with brain abscess via neuroimaging examinations, at which point CSF puncture was no longer clinically meaningful and carried an extremely high risk.

**Table 2:** Biochemical identification result.

Strain	Indole	Lactose	Esculin	Mannitol	Glucose	Sorbitol	Salicin	Maltose
*F.n* standard strain	(+)	(-)	(-)	(-)	(-)	(-)	(-)	(-)
*F.n* clinical strain	(+)	(-)	(-)	(-)	(-)	(-)	(-)	(-)

(+) is positive, (-) is negative.

### 3.2 Analysis of biological characteristics of the *F.n* clinical strain

#### 3.2.1 Colony and morphological characteristics

Colonies of the *F.n* clinical strain were milky white with smooth edges, and the diameter was approximately 0.5-1mm (**Figure 1K**). Under light microscopy, the clinical strain appeared as slender bacilli with tapered ends and filamentous structures (**Figure 1M**). Both colony morphology and microscopic morphology were similar to those of the F.n standard strain (**Figures 1J, L**).

#### 3.2.2 Growth kinetics and biofilm formation characteristics

The bacterial growth curve showed that in the liquid medium, the growth rate of the *F.n* clinical strain was slightly lower than that of the standard strain. But the final reproduction was almost the same (**Figure 1N**).

The biofilm curves showed that the biofilm formation process was similar between two strains. The amount of biofilm all peaked at18 h and then decreased slightly. But the final biofilm formation of the *F.n* clinical strain was lower than that of the standard strain (**Figure 1O**).

### 3.3 Drug susceptibility results

**Figure 2:**
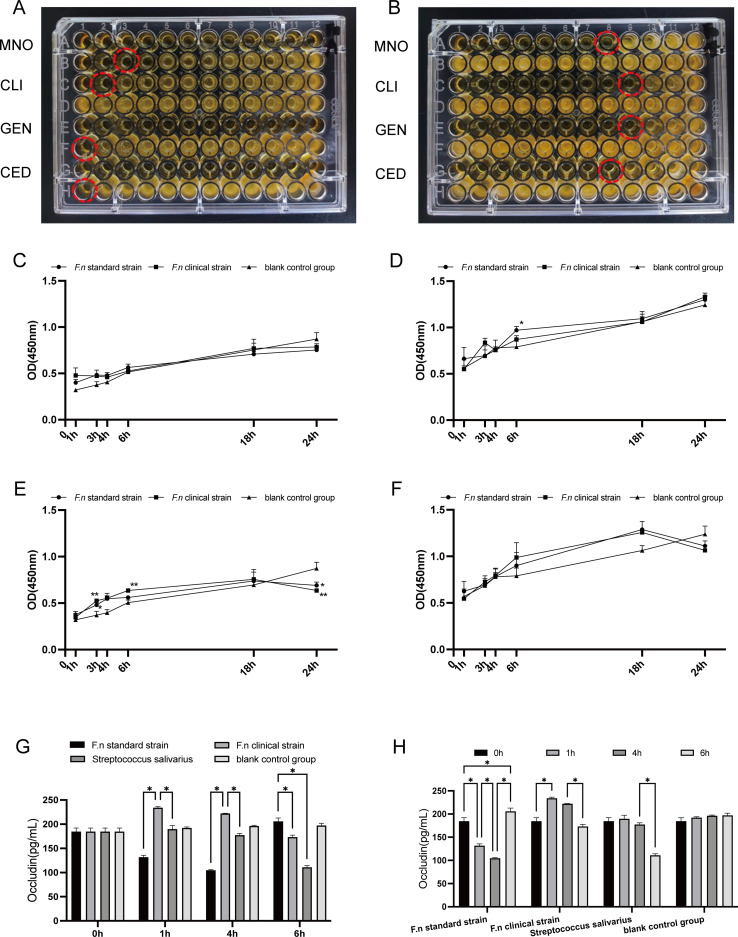
**(A)** MIC assay of *F.n* standard strain. **(B)** MIC assay of *F.n* clinical strain. Red circles indicate the well corresponding to the minimum inhibitory concentration (MIC). **(C)***F.n* infected KB cells, MOI = 10:1. **(D)***F.n* infected bEnd.3 cells, MOI = 10:1. **(E)***F.n* infected KB cells, MOI = 100:1. **(F)***F.n* infected bEnd.3 cells, MOI = 100:1. One-way ANOVA compared the difference between groups at different time points, Dunnett t-test compared the difference between the experimental group and the blank control group at different time points, *means *p* < 0.05 compared with the blank control group, **means *p* < 0.01 compared with the blank control group. **(G, H)** Expression of Occludin. *means *p* < 0.05.

The minimum inhibitory concentrations (MIC) of the four drugs against *F.n* standard and clinical strains were shown (**Table 3**). The MIC of minocycline, gentamicin and cephradine against the standard strain was lower than the MIC of the clinical strain. On the contrast, the MIC of clindamycin against the standard strain was higher than the MIC of the clinical strain. Among the four drugs tested, minocycline had the lowest MIC against *F.n* standard strain, while the MIC of clindamycin was the lowest against the *F.n* clinical strain (**Figures 2A, B**).

**Table 3:** The minimum inhibitory concentration of *F.n* standard strain and *F.n* clinical strain (μg/mL).

Antibiotic	*F.n* standard strain	*F.n* clinical strain
MNO	0.03	1.95
CLI	62.5	0.98
GEN	6.10	97.66
CED	0.61	19.53

Minimum inhibitory concentration (MIC) values represented the exact values determined by the probit method specified in the guidelines of the Clinical and Laboratory Standards Institute (CLSI).

The antibacterial ring diameter of minocycline, gentamicin and cephradine against *F.n* standard strain was larger than that against *F.n* clinical strain. Moreover, the antibacterial ring diameter of clindamycin against the *F.n* clinical strain was larger than that against standard strain (**Table 4**; **Figures 1P, Q**).

**Table 4:** Antibacterial ring diameter of *F.n* standard strain and *F.n* clinical strain (mm, $\bar{x}$ ± s).

Antibiotic	*F.n* standard strain	*F.n* clinical strain	t	*p*
MNO(25μg/mL)	19.33 ± 2.05	18.50 ± 1.47	0.466	0.665
CLI(25μg/mL)	16 ± 1.63	23 ± 0.82^*^	4.490	0.011
GEN(25mg/mL)	18.83 ± 1.03	12.33 ± 1.25^*^	4.111	0.014
CED(2.5mg/mL)	18.67 ± 1.70	17.67 ± 1.70	0.588	0.588

*Student's t test, compared with *F.n* standard strain,*p* < 0.05.

### 3.4 Effects of *F.n* on the proliferation of KB cells and bEnd.3 cells

At low multiplicity of infection (MOI = 10), both strains showed no significant inhibition of cell proliferative capacity within 24h. At higher multiplicity of infection (MOI = 100), both strains appeared to inhibit cell proliferation (**Figures 2C–F**).

### 3.5 Adhesion and invasion ability of *F.n* to KB cells and bEnd.3 cells

Both the standard strain and clinical strain exhibited the ability to adhere to and invade KB cells and bEnd.3 cells. Further comparison showed that the standard strain exhibited significantly higher adhesion and invasion abilities than the clinical strain. In addition, in both cell models, the standard strain and clinical strain showed higher invasion ability toward bEnd.3 cells than toward KB cells. Details are shown in **Table 5**.

**Table 5:** The adhesion (%, x̄ ± s) and invasion ability (‰, x̄ ± s) to KB and bEnd.3 cells.

	Adhesion ability		Invasion ability	
Cell line	*F.n* standard strain	*F.n* clinical strain	*F.n* standard strain	*F.n*clinical strain
KB	0.92 ± 0.20	0.61 ± 0.15^c^	0.45 ± 0.14	0.35 ± 0.07^f^
bEnd.3	0.98 ± 0.24^a^	0.75 ± 0.16^bd^	0.53 ± 0.08^e^	0.39 ± 0.10^g^

Poisson distributed independent sample z test.^a, b^Compared with KB group, *p* < 0.01.^c, d^Compared with *F.n* standard strain,*p* < 0.01. Poisson distribution independent sample z test.^e^Compared with KB group,*p* < 0.05, ^f^Compared with *F.n* standard strain,*p* < 0.01, ^g^Compared with *F.n* standard strain,*p* < 0.01.

### 3.6 Expression of tight junction proteins in brain microvascular endothelial cells

#### 3.6.1 Establishment of the *in vitro* model and verification of barrier function

The TEER value of the model was stably maintained at 15-35 Ω·cm², and\ the permeability coefficient of sodium fluorescein was approximately 4.0 × 10^–6^ cm/s, indicating that the *in vitro* model possessed intact and reliable barrier function.

#### 3.6.2 Expression of occludin

The expression in the *F.n* standard strain group showed a trend of first down-regulation followed by up-regulation. In the clinical strain group, the expression exhibited a trend of initial increase and subsequent decrease. The differences in protein expression levels at each time point were statistically significant (*p* < 0.05) (**Figure 2G**).

At 1 h and 4 h, the expression in the clinical strain group was significantly higher than that in standard strain group (*p* < 0.05) and the Streptococcus salivarius group (*p* < 0.05) (**Figure 2H**). All differences were statistically significant.

#### 3.6.3 Expression of Claudin-5

**Figure 3:**
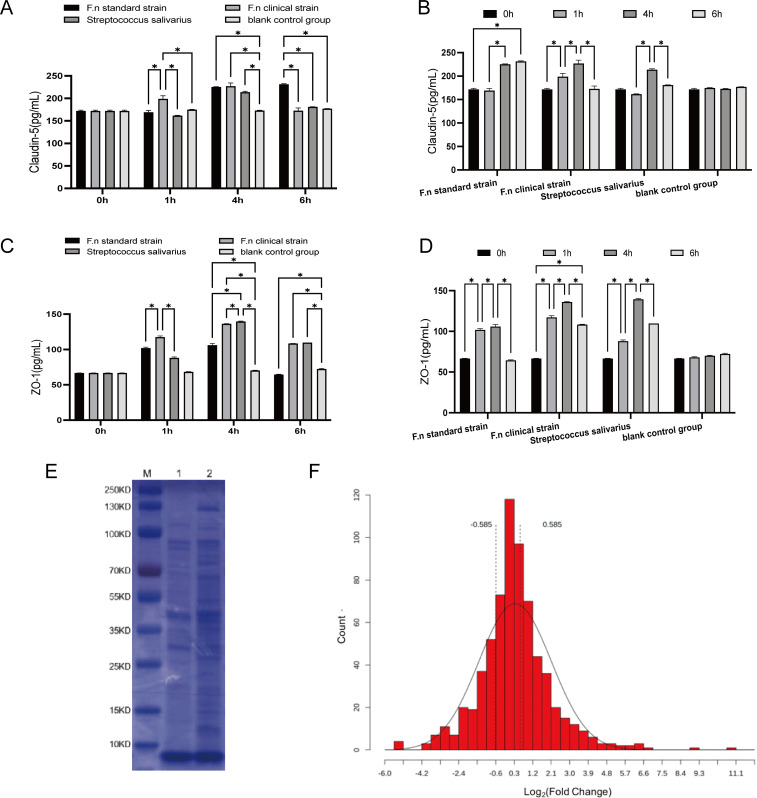
**(A, B)** Expression of Claudin-5. * means *p* < 0.05. **(C, D)** Expression of ZO-1. * means *p* < 0.05. **(E)** SDS-PAGE gel electrophoresis of *F.n* standard strain and *F.n* clinical strain. M: Marker, 1: *F.n* standard strain, 2: *F.n* clinical strain. **(F)** Histogram of the distribution of protein quantitative ratios. Note: The dashed line indicates the quantitative ratio threshold standard for screening differential protein selection, and the differential protein is outside the two dashed lines.

The expression in the *F.n* standard strain group showed a continuous upward trend, while that in the clinical strain group exhibited a trend of initial increase followed by decrease (**Figure 3A**).

At 1 h, Claudin-5 expression in the clinical strain group was significantly higher than that in all other groups (*p* < 0.05). At 4 h, the expression level was comparable between the standard and clinical strain group. At 6 h, Claudin-5 expression in the standard strain group remained higher than that in all other groups (*p* < 0.05), whereas the levels in the clinical strain group and the Streptococcus salivarius group decreased to nearly those of the blank control group (**Figure 3B**). All differences were statistically significant.

#### 3.6.3 Expression of ZO-1

ZO-1 expression was elevated at an early time point and then decreased gradually in both the standard strain group and the clinical strain group (**Figure 3C**).

At 1 h, ZO-1 expression in the clinical strain group was significantly higher than that in the standard strain group (*p* < 0.05) and the Streptococcus salivarius group (*p* < 0.05). At 6 h, ZO-1 expression in the clinical strain group and the Streptococcus salivarius group was higher than that in the blank control group (*p* < 0.05), while that in the standard strain group was lower than that in the blank control group (**Figure 3D**). All differences were statistically significant.

### 3.7 Proteomic differential analysis

#### 3.7.1 SDS-PAGE gel electrophoresis

The clinical strain and the standard strain shared similar protein bands while also displaying distinct differential bands (**Figure 3E**).

#### 3.7.2 Screening of differentially expressed proteins

A total of 688 proteins were identified, among which 654 could be quantified (**Table 6**). Compared with *F.n* standard strain, clinical strain had 171 significantly up-regulated proteins, 114 significantly down-regulated proteins, 73 proteins unique to clinical strain, and 39 proteins unique to standard strain (**Tables 7**, **8**). The distribution of the differential proteins is shown in **Figure 3F**.

**Table 6:** Number of proteins and peptides identified.

Analytical category	*F.n* standard strain	*F.n* clinical strain	Total
Identified proteins	623	649	688
Quantified proteins	591	622	654
Identified peptides	3270	3480	3987
Unique peptides	3250	3456	3963

**Table 7:** The number of differentially expressed proteins.

Category	*+*	-	Total difference	No difference	Total
Number	244	153	397	257	654

"+" indicates a significantly higher quantification in the clinical strain; "-" indicates a significantly higher quantification in the standard strain,*p* < 0.05.

**Table 8:** Subdivision of differential proteins.

Category	Proteins of *F.n* standard strain		Proteins of *F.n* clinical strain	
	Significantly upregulated	Specifically expressed	Significantly upregulated	Significantly upregulated
Number	114	39	171	73
Total	153		244	

#### 3.7.3 Functional annotation and pathway enrichment

**Figure 4:**
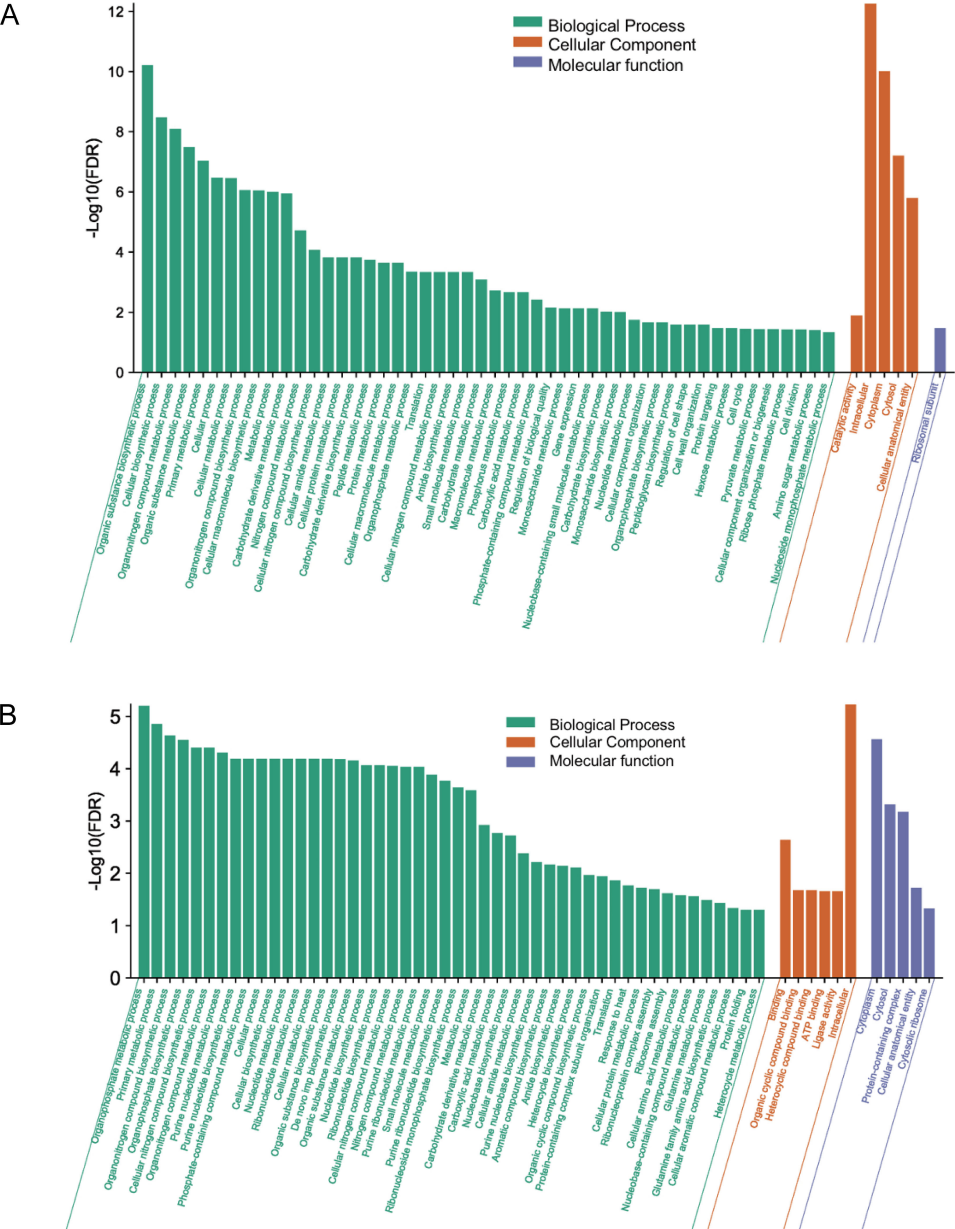
**(A)** GO enrichment analysis results of upregulated differentially expressed proteins. **(B)** GO enrichment analysis results of downregulated differentially expressed proteins.

Among the biological processes involving upregulated differential proteins, those with significant enrichment included the biosynthesis of organic substances, biosynthesis of macromolecules, cell cycle, and protein metabolism. Among molecular functions, catalytic activity showed significant enrichment. Among cellular components, those with significant enrichment included cytoplasm and ribosomal subunits (**Figure 4A**).

Among the biological processes involving downregulated differential proteins, those with significant enrichment included the metabolism and synthesis of organic phosphates, metabolism and synthesis of organic nitrogen compounds, and metabolism and synthesis of nucleotides and ribonucleotides. Among molecular functions, those with significant enrichment included ATP binding and ligase activity. Among cellular components, those with significant enrichment included cytoplasm, cell membrane, and cytoplasmic ribosomes (**Figure 4B**).

#### 3.7.4 Key differentially expressed proteins

FC stands for Fold Change, which is calculated as the ratio of the protein expression level of the clinical strain to that of the standard strain [FC = Protein expression level of *F.n* clinical strain/Protein expression level of *F.n* standard strain].

The top 3 most significantly up-regulated proteins in clinical strain were Amidinotransferase (FC = 385.429), Uracil-DNA glycosylase (FC = 197.837), and DUF3826 domain-containing protein (FC = 86.350) (**Table 9**).

**Table 9:** Up-regulated differential proteins in the *F.n* clinical strain.

Name	Gene	FC	Function
Amidinotransferase	HMPREF9094_2384	385.429	Involve in the metabolism of guanidine-containing compounds
Uracil-DNA glycosylase	Ung	197.837	Involve in the process of DNA damage repair
DUF3826 domain-containing protein	HMPREF9094_1627	86.350	Not clear yet
Phosphoglycerate mutase	Pgm2	39.796	Key enzymes in the glycolytic pathway
Fe³^+^ ABC superfamily ATP binding cassette transporter	HMPREF9094_1549	25.158	Transport of iron ions
Flavodoxin	FprA3	22.789	Involve in electron transport
DUF4265 domain - containing protein	HMPREF9094_2465	19.770	Not clear yet
Membrane protein	HMPREF9094_1900	18.289	Substance transport; signal transduction; cell adhesion
Pyruvate formate-lyase activating enzyme	PflA	15.890	Involve in anaerobic metabolism
GTPase Der	EngA	15.687	Involve in the division and proliferation of cells

Top ten up-regulated proteins with the largest difference.

The top 3 most significantly down-regulated proteins were Co-chaperonin GroES (FC = 0.00169), C4-dicarboxylate - binding protein (FC = 0.02500), and Peptidase T (FC = 0.03101) (**Table 10**).

**Table 10:** Down-regulated differential proteins in the *F.n* clinical strain.

Name	Gene	FC	Function
Co-chaperonin GroES	groES	0.00169	Help other proteins fold correctly
C4-dicarboxylate - binding protein	HMPREF9094_0945	0.02500	Involve in the transport and metabolism of dicarboxylic acids
Peptidase T	HMPREF9094_1629	0.03101	Hydrolyze peptide bonds and participate in the degradation of proteins
Dicarboxylate:H^+^ TRAP - T family tripartite ATP - binding transport system	HMPREF9094_0946	0.03333	Involve in the transport of dicarboxylic acid and other substances
Hydroxymethylbilane synthase	HemC	0.04487	Involve in the porphyrin biosynthesis pathway
MORN repeat protein	HMPREF9094_0180	0.05208	Involve in the interaction between proteins and membranes and intracellular signaling
5-nitroimidazole antibiotic resistance protein	HMPREF9094_0119	0.05570	Relate to the resistance of bacteria to 5-nitroimidazole antibiotics
Lipoprotein	HMPREF9094_2396	0.05917	Involve in adhesion, signal transduction, material transport and other processes
Purine nucleoside phosphorylase	HMPREF9094_0616	0.05928	Involve in purine metabolic pathways
NhaC family sodium:proton (Na^+^:H^+^) antiporter	HMPREF9094_0230	0.06085	Regulate sodium ion and proton concentration balance

Top ten down-regulated proteins with the largest difference.

#### 3.7.5 Differential expression of proteins related to immune escape and drug resistance

Differentially expressed proteins associated with immune escape and antibiotic resistance are shown in **Table 11**. TraT complement resistance protein was significantly upregulated in the clinical strain. 5-nitroimidazole antibiotic resistance protein and RND superfamily resistance-nodulation-cell division antiporter were downregulated in the clinical strain (**Table 11**).

**Table 11:** Differentially expressed proteins associated with immune escape and antibiotic resistance.

Name	Gene	FC
TraT complement resistance protein	TraT	3.27449
5 - nitroimidazole antibiotic resistance protein	HMPREF9094_0119	0.05570
RND superfamily resistance-nodulation-cell division antiporter	HMPREF9094_1811	0.76387

FC means Fold Change (*F.n* clinical strain/*F.n* standard strain).

#### 3.7.6 Differential proteins associated with the ribosomal 50S subunit

50S ribosomal protein L16, L9 and L30 were significantly upregulated in the clinical strain (**Table 12**).

**Table 12:** Differential proteins associated with the ribosomal 50S subunit in *F.n* clinical strains.

Name	Gene	FC
50S ribosomal protein L16	rplP	2.0303
50S ribosomal protein L9	rplI	2.06120
50S ribosomal protein L30	rpmD	1.62560
50S ribosomal protein L5	rplE	0.49184
50S ribosomal protein L11	rplK	0.21018

#### 3.7.7 Differential expression of outer membrane channel-related proteins

ABC transporter binding protein and outer membrane protein TolC were upregulated in the clinical strain (**Table 13**).

**Table 13:** Differential expression of outer membrane channel-related proteins.

Name	Gene	FC	Function
Outer membrane protein TolC	tolC2	3.72246	Facilitates the extrusion of toxic substances and antimicrobial agents, playing a role in multidrug resistance.
Outer membrane protein TolC	tolC3	2.50792	Facilitates the extrusion of toxic substances and antimicrobial agents, playing a role in multidrug resistance.
ABC transporter binding protein	HMPREF9094_1003	10.0568	Recognizes and binds specific nutrients to initiate their transmembrane transport.
Outer membrane protein	HMPREF9094_0645	10.1512	Involved in substance transport or signal transduction.
Outer membrane protein P1	HMPREF9094_0535	7.82926	Functions as a selective channel or adhesin, mediating bacterium-host interactions.

#### 3.7.8 KEGG pathway

**Figure 5:**
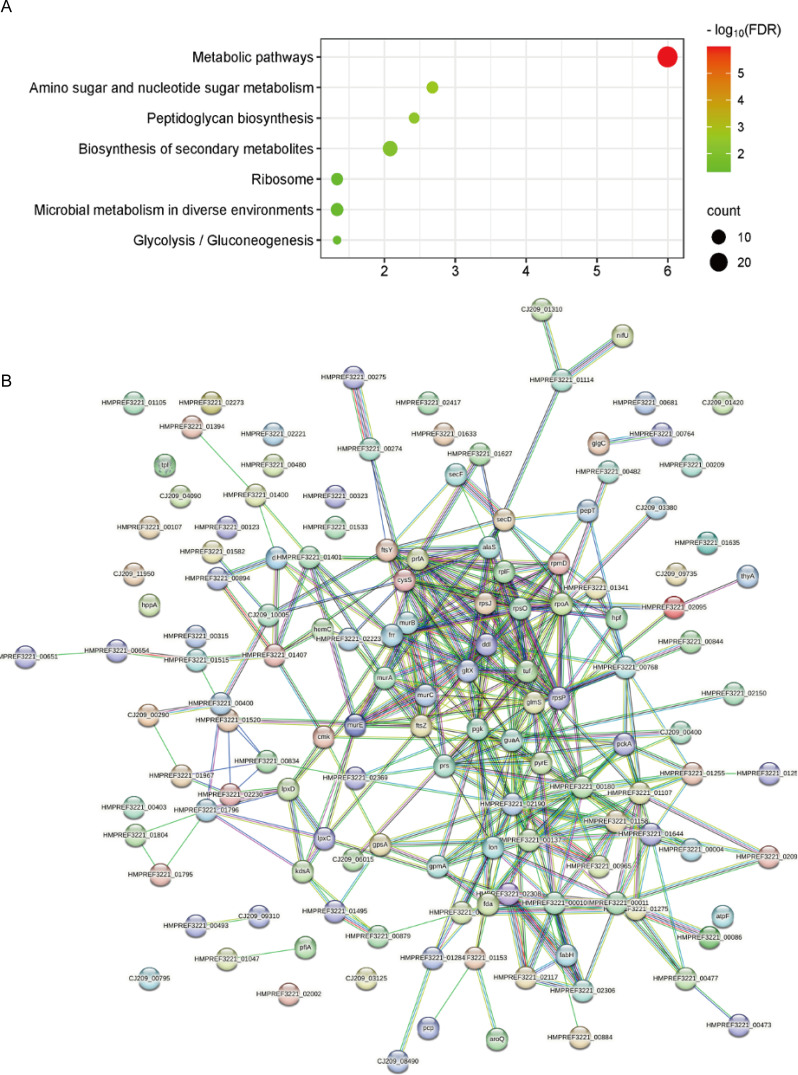
**(A)** KEGG pathway enrichment analysis results of upregulated proteins. **(B)** STRING protein-protein interaction network of upregulated proteins, *p* = 1.21e-07.

The KEGG pathways with significant enrichment of upregulated differential proteins included Amino Sugar and Nucleotide Sugar Metabolism, Peptidoglycan Biosynthesis, and Glycolysis/Gluconeogenesis (**Figure 5A**).

**Figure 6:**
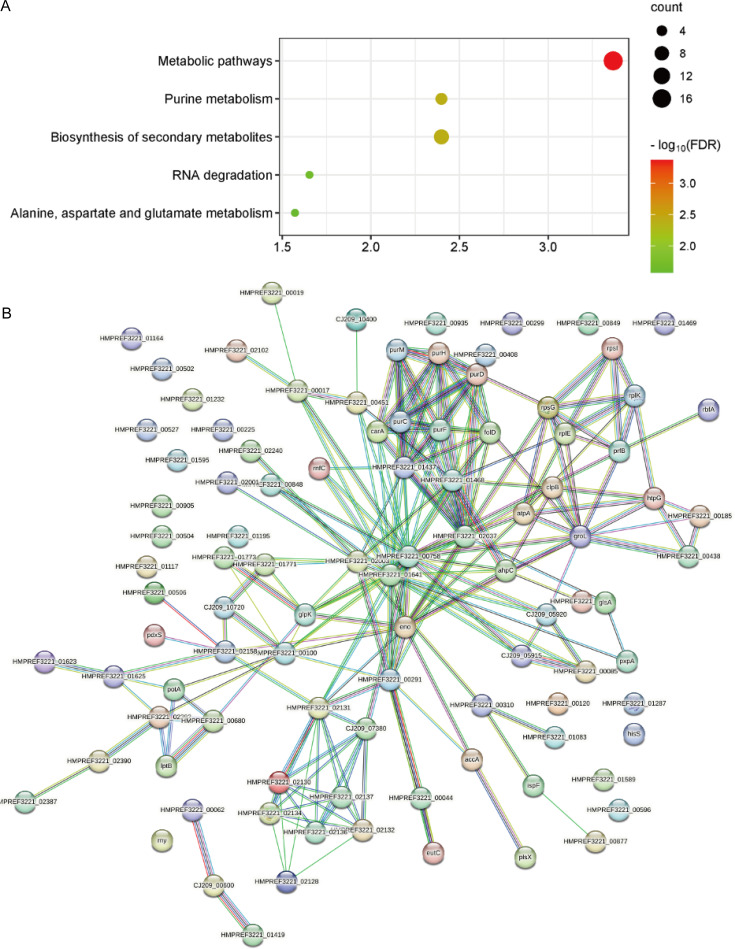
**(A)** KEGG pathway enrichment analysis results of downregulated proteins. **(B)** STRING protein-protein interaction network of downregulated proteins, *p* = 2.31e-08.

The KEGG pathways with significant enrichment of downregulated differential proteins included Purine Metabolism, RNA Degradation, and Alanine, Aspartate and Glutamate Metabolism (**Figure 6A**).

Protein-protein interaction (PPI) network analysis of the proteins was performed using the STRING database. The p-value for PPI enrichment of upregulated differential proteins was 2.46e-09, and the p-value for PPI enrichment of downregulated differential proteins was 2.31e-08. These results indicate that these proteins are functionally interconnected in biological processes (**Figures 5B**, **6B**).

## 4 Discussion

This study focused on the *F.n* standard strain ATCC 25586 and a clinical strain isolated from brain abscess as the research subjects. Through strain identification, analysis of biological characteristics, bacterial-cell interaction experiments, antimicrobial susceptibility testing, and proteomic analysis, the potential mechanisms by which *Fusobacterium nucleatum* crosses the blood-brain barrier were preliminarily explored at multiple levels.

### 4.1 Adhesion and invasion ability of *Fusobacterium nucleatum* to brain microvascular endothelial cells

Bacterial adhesion to and invasion of host cells represent crucial initial steps in the infection process (Drevets et al., 2004; Krachler and Orth, 2013; Kemper and Hensel, 2023). The brain microvascular endothelial cell (BMEC) layer within the blood-brain barrier serves as the primary defense of the central nervous system, and its integrity directly determines whether pathogens can breach the intracranial space (Daneman and Prat, 2015). Compared to oral epithelial cells, *Fusobacterium nucleatum* demonstrated significantly stronger adhesion and invasion capabilities against BMECs. This difference suggests that BMECs may provide a more favorable interface for bacterial attachment. Unlike stratified epithelial cells with dense keratinized layers, the defense of the monolayer endothelial barrier formed by BMECs relies on highly sophisticated and dynamically regulated intercellular junctions (Matter and Balda, 2007). These junctions can undergo remodeling in response to physiological and pathological signals (Keaney and Campbell, 2015; Stamatovic et al., 2016). This inherent “plasticity” of junctions, which exists to adapt to the intracranial microenvironment, may offer *Fusobacterium nucleatum* a more compatible interaction interface and potential opportunities for invasion.

Such cell type-dependent differences in adhesion and invasion may be related to the specific recognition between bacterial adhesins and host cell surface receptors. It is known that endothelial cells express various adhesion molecules (such as ICAM-1, VCAM-1), which are upregulated under inflammatory conditions and often serve as ‘anchoring points’ for pathogen invasion (Pober and Sessa, 2007; Konradt and Hunter, 2018; Guerra-Espinosa et al., 2024). Specific receptors or molecular interaction interfaces that are more readily recognized and bound by *F.n* may exist on the surface of brain microvascular endothelial cells.

Although phenotypic differences were observed in this study, it should be noted objectively that the oral epithelial cells used were of human origin, while the brain microvascular endothelial cells were of mouse origin. Due to the species-dependent differences in the inherent affinity of *F.n* for human and mouse cells, the results of this study only reflect the preliminary *in vitro* trend of its invasion into brain microvascular endothelial cells. The applicability of these conclusions to the human physiological microenvironment remains to be further verified using human brain microvascular endothelial cells, organoid models, or more clinically relevant animal models.

Meanwhile, the present study determined the non-cytotoxic multiplicity of infection (MOI) via CCK-8 assay, eliminating the interference of cell damage on adhesion and invasion experiments. Under this condition, we found that the adhesive and invasive capacities of the clinical strain toward both cell types were significantly weaker than those of the standard strain. However, growth curve analysis showed no significant difference in proliferative capacity between the two strains. Combined with the upregulation of metabolism-related proteins in the clinical strain revealed by subsequent proteomic analysis, we speculate that this difference does not simply represent reduced virulence, but may be related to the adaptation of the strain to distinct microenvironments. The standard strain exhibits stronger adhesion and invasion ability, which is more conducive to breaching the host barrier. In contrast, the clinical strain was isolated from an intracranial abscess lesion, and its phenotype of lower adhesion and invasion ability accompanied by upregulated metabolism-related proteins may represent an adaptive alteration to the intracranial microenvironment.

### 4.2 Differential regulation of tight junction proteins in brain microvascular endothelial cells by *Fusobacterium nucleatum*

The functional integrity of the blood–brain barrier is highly dependent on the structure and dynamic regulation of tight junctions between brain microvascular endothelial cells. Among them, the transmembrane protein Claudin-5 serves as the core “seal” for forming the paracellular barrier selectivity (Greene et al., 2019). As a key regulatory protein, the phosphorylation status of occludin is directly involved in the dynamic regulation of tight junction permeability (Raleigh et al., 2011). In contrast, the intracellular scaffolding protein ZO-1 anchors transmembrane proteins to the cytoskeleton and acts as a structural hub for maintaining tight junction stability (Stamp et al., 2023). Changes in the expression levels and distribution of these three proteins are classic molecular markers for evaluating the functional status of the blood–brain barrier (Dithmer et al., 2024). Based on this, the present study monitored the dynamic expression of the above-mentioned proteins after co-culturing the *in vitro* bEnd.3 monolayer cell model with *F.n* via quantitative ELISA. To distinguish specific pathogenic effects from general stimulation by microbe-associated molecular patterns (MAMPs), a non-invasive Streptococcus salivarius strain was specifically introduced as a negative control in the experiment.

This study found that *F.n* can regulate the expression of tight junction proteins in brain microvascular endothelial cells, and the regulatory patterns differ between the standard strain and the clinical strain. This may represent one of the potential molecular mechanisms through which it promotes blood-brain barrier dysfunction. Notably, unlike the changes observed in Occludin and ZO-1, both bacterial strains consistently induced upregulation of Claudin-5 expression. This finding differs from the conventional view that inflammation universally disrupts tight junctions. We speculate that Claudin-5, as a core protein maintaining the selectivity of the blood-brain barrier, may undergo compensatory upregulation as an adaptive or compensatory response of endothelial cells during the early stages of infection (Wang et al., 2023; Örüm et al., 2025). The underlying regulatory mechanism warrants further in-depth investigation.

Significant differences were observed in the time-course regulatory patterns of tight junction proteins between the standard strain and the clinical strain. The standard strain induced fluctuating changes in the expression of Occludin and ZO-1, and it exhibited strong adhesive and invasive capacities. This may result from the influence of direct cell−cell interactions between bacteria on the homeostasis of junctional proteins. Although the clinical strain exhibits weaker adhesion and invasion, it exerts a more potent and sustained effect on the expression of tight junction proteins. This strain may modulate endothelial cells via the secretion of soluble factors. The detailed regulatory mode and underlying molecular mechanisms warrant further investigation.

It is worth noting that although the non-pathogenic Streptococcus salivarius (serving as the negative control) also induced time-dependent changes in tight junction proteins, the magnitude of these changes was weaker than that induced by *F.n*, and no irreversible damage to the barrier function was caused. This most likely represents a basic and universal immune surveillance and adaptive barrier regulation of microorganisms by host brain microvascular endothelial cells through pattern recognition receptors. In contrast, the severe and persistent protein expression dysregulation and functional impairment induced by *F.n* far exceeded the scope of this general immune surveillance response. This further confirms the specificity and definite pathogenicity of the regulatory effect of *F.n*.

### 4.3 Efficient adaptation of clinical strain to the intracranial microenvironment via proteomic reprogramming

Proteomic analysis revealed that the expression of 285 proteins was altered in the clinical strain. The upregulation of metabolism-related proteins may facilitate its adaptation to the hypoxic and nutrient-limited microenvironment of intracranial abscess. The altered expression of immunity-related proteins may contribute to evading or resisting immune pressure in the intracranial cavity. Such reprogramming of protein expression provides molecular support for the adaptation of the clinical isolate to the intracranial microenvironment.

#### 4.3.1 Metabolic pathway remodeling optimizes intracranial nutrient utilization and energy metabolism

Our results showed that upregulated proteins in the clinical strain were significantly enriched in pathways including amino sugar and nucleotide sugar metabolism, peptidoglycan biosynthesis, and glycolysis/gluconeogenesis. The most striking finding was that amidinotransferase was extremely upregulated in the clinical strain (with a fold change as high as 385.4), while its expression level was extremely low in the standard strain. This hundreds−fold difference in expression suggests that the intracranial abscess microenvironment may exert a selective pressure on the metabolic pathways of *Fusobacterium nucleatum*, which may be critical for the survival of the clinical strain in the brain. By upregulating the expression of this key enzyme, the clinical strain may optimize its utilization of nutrients in the intracranial microenvironment, thereby providing a metabolic advantage for its colonization and survival.

With regard to energy metabolism, enrichment of the glycolysis/gluconeogenesis pathway implies that the clinical strain adapts to the hypoxic environment by enhancing anaerobic respiration. This is consistent with the growth curve results showing that its growth rate was slower, but the final biomass was comparable to that of the standard strain. The low energy yield from anaerobic respiration may limit its growth rate, and this phenotype may represent an adaptive response of the clinical strain to the hypoxic intracranial microenvironment. Downregulation of pathways such as purine metabolism may allow the clinical strain to reduce its proliferative metabolic activity andconserve energy, thereby favoring its survival in the intracranialmicroenvironment. The above changes in the expressionofmetabolism-related proteins represent metabolic adaptive characteristics of the clinical strain to the intracranial microenvironment.

#### 4.3.2 Upregulation of immune escape-related proteins enhances resistance to intracranial immune pressure

The intracranial cavity harbors a highly activated immune surveillance network composed of microglia, astrocytes, and the complement system, which exerts strong innate immune selective pressure (Jackson et al., 2025; Müller et al., 2025). In the clinical strain, the TraT complement resistance protein was significantly upregulated. This protein can evade elimination by the host innate immune system by inhibiting the complement cascade, thereby providing immune evasion support for its survival in the intracranial cavity. Meanwhile, the expression of UDP-3-O-acylglucosamine deacetylase, a key enzyme involved in lipopolysaccharide synthesis, was also upregulated in the clinical strain, which may modulate the local immune microenvironment by regulating the immunostimulatory effects of endotoxins (FC = 2.67146). Its specific function and association with blood–brain barrier function and bacterial colonization warrant further investigation.

#### 4.3.3 Changes in the drug resistance spectrum of clinical strains are associated with differential proteomic expression

Antimicrobial susceptibility testing results showed that the clinical strain had increased sensitivity to clindamycin, but decreased sensitivity to minocycline, gentamicin, and cefradine. The aforementioned proteomic analysis provided an explanation for this phenotypic difference. Clindamycin inhibits protein synthesis by binding to the 50S ribosomal subunit, and the significant downregulation of 50S ribosomal subunit-related proteins in the clinical strain may have altered the conformation or affinity of the drug target, thereby increasing its sensitivity. In contrast, the decreased sensitivity of the clinical strain to the other three antibiotics may be closely related to the remodeling of its transporter system. The significant upregulation of the Fe³^+^ ABC superfamily ATP binding cassette transporter (FC = 25.158) not only enhances its key ability to uptake iron in the intracranial environment but also its inherent efflux pump function may directly reduce intracellular antibiotic concentrations. In addition, In addition, the upregulation of the outer membrane channel protein TolC, as a common channel for various efflux pumps, its high expression is not only associated with a multidrug-resistant phenotype but also suggests that the clinical strain may possess a more active virulence factor secretion or quorum sensing system, which is consistent with its overall evolution toward a “remote-regulation” pathogenic strategy. It is worth mentioning that, guided by the antimicrobial susceptibility results from this experiment, a clindamycin treatment regimen was formulated for the patient, which achieved significant therapeutic outcomes including symptom relief and abscess reduction.

### 4.4 Combined consideration of the blood-brain barrier and blood-cerebrospinal fluid barrier

Based on the *in vitro* bEnd.3 cell model, this study focused on investigating the adhesion, invasion, and tight junction regulatory mechanisms of *F.n* on the BBB. In addition, the central nervous system defense barrier also includes the BCSFB composed of choroid plexus epithelial cells, which is also an important portal for pathogenic intracranial invasion. The clinical data of this study (**Table 1**) showed abnormal cerebrospinal fluid indicators in patients with early intracranial abscesses, suggesting that their BCSFB has undergone functional impairment and indicating that the central nervous system invasion of *F.n* may not be a single barrier pathway. In the future, a BCSFB model containing choroid plexus epithelial cells can be established to explore the strategies and timing of *F.n* crossing different central nervous system barriers, thereby further improving the elaboration of its pathogenic mechanism in intracranial infection.

## 5 Conclusion

This study mainly investigated the mechanisms underlying blood–brain barrier penetration and intracranial colonization of *Fusobacterium nucleatum* from the perspective of pathogen adaptive evolution. *Fusobacterium nucleatum* can adapt to different environments by altering biological characteristics such as adhesion, invasion, and biofilm formation, as well as regulatory patterns of tight junction proteins. In addition, it can reshape metabolic pathways to enhance anaerobic respiration and optimize nutrient utilization, thereby adapting to the hypoxic and nutrient-limited intracranial microenvironment. Concurrently, upregulation of immune evasion-related proteins strengthens resistance to intracranial immune pressure, conferring certain survival advantages in the brain.

## Data Availability

The original contributions presented in the study are publicly available. The mass spectrometry proteomics data have been deposited to the ProteomeXchange Consortium (https://proteomecentral.proteomexchange.org via the iProX partner repository with the dataset identifier PXD076046.
